# Polycomb Group Proteins in *Drosophila melanogaster*

**DOI:** 10.3390/biology15171470

**Published:** 2026-08-31

**Authors:** Fei Gao, Zicheng Li, Xiaoping Yu, Sunjie Chen, Chengcheng Ye

**Affiliations:** 1 Key Laboratory of Microbiological Metrology, Measurement & Bio-Product Quality Security, State Administration for Market Regulation, China Jiliang University, Hangzhou 310018, China; gaofei@cjlu.edu.cn (F.G.); yxp@cjlu.edu.cn (X.Y.); 2 Wenzhou Plant Protection and Soil Fertilizer Management Station, Wenzhou 325000, China; lizicheng386@zju.edu.cn

**Keywords:** Polycomb group, *Drosophila melanogaster*, epigenetics, development, transcriptional regulation

## Abstract

The development of multicellular organisms relies on the precise establishment and stable inheritance of cell type-specific transcriptional programs without changes to the underlying DNA sequence. Polycomb group (PcG) proteins are evolutionarily conserved epigenetic repressors that govern developmental gene expression by assembling into multiprotein complexes to establish robust and spatiotemporally restricted transcriptional silencing. Since their discovery through the classic Polycomb mutant phenotype in *Drosophila melanogaster*, PcG proteins have become a central paradigm for understanding epigenetic regulation during animal development. Here, we synthesize current knowledge of the molecular composition and functional organization of the core Polycomb repressive complexes (PRC1 and PRC2) and the accessory complexes PhoRC, PR-DUB, and dRAF, with particular emphasis on canonical hierarchical recruitment and emerging noncanonical regulatory mechanisms of Polycomb-mediated gene repression. We further summarize the dynamic roles of PcG proteins across the *Drosophila* life cycle, from embryonic patterning and larval organogenesis to metamorphic transition and adult tissue homeostasis. By integrating recent advances from *Drosophila* studies, this review provides a comprehensive overview of the molecular mechanisms and developmental functions of PcG proteins while highlighting future directions for investigating the conserved epigenetic mechanisms governing metazoan development.

## Simple Summary

During development, cells must preserve their specific identities while continuously dividing and responding to environmental changes. This stability is maintained by epigenetic regulation, a system that controls gene activity without changing DNA sequences. Polycomb group (PcG) proteins are essential epigenetic regulators that form specialized protein complexes to maintain the silencing of key developmental genes and ensure stable cell identity. This review summarizes the composition and functions of five major PcG complexes in *Drosophila*, describing both classical mechanisms of gene repression and recently discovered non-canonical regulatory modes. We also discuss how PcG proteins regulate biological processes throughout the *Drosophila* life cycle, including embryonic development, larval growth, metamorphosis, adult reproduction, ageing and environmental adaptation. Research on PcG proteins in *Drosophila* has established fundamental concepts in epigenetics and continues to provide valuable insights into human disease mechanisms and environmentally friendly approaches for insect pest control.

## 1. Introduction

The development of multicellular animals begins with a single fertilized egg and culminates in the formation of a complex organism composed of diverse tissues and organs. This remarkable process relies on the precise spatiotemporal regulation of developmental gene expression, whereby lineage-specific genes are activated in the appropriate cellular and developmental contexts while being stably repressed in inappropriate contexts. Such coordinated regulation ensures accurate cell fate specification and the orderly progression of tissue and organ morphogenesis [1,2]. A fundamental feature of this regulatory network is the contribution of epigenetic mechanisms, which confer heritable control of gene expression without altering the underlying DNA sequence. By remodeling chromatin organization and regulating chromatin accessibility, epigenetic pathways establish and preserve transcriptional states across cell divisions, thereby providing a conserved molecular basis for developmental regulation throughout metazoan evolution [3,4].

Among the major epigenetic regulators, Polycomb group (PcG) proteins constitute a conserved transcriptional repression system that preserves developmental gene silencing and cellular identity. PcG components are present throughout metazoans, from *Drosophila* to mammals, reflecting strong evolutionary conservation of this regulatory machinery [5,6,7,8,9,10,11,12]. However, rather than representing a fixed repression module, PcG proteins have undergone extensive diversification through the assembly of distinct multiprotein complexes and context-dependent regulatory modules, allowing flexible control of developmental programs in different biological settings [13,14,15]. The core Polycomb machinery comprises two evolutionarily conserved multisubunit complexes, Polycomb Repressive Complex 1 (PRC1) and Polycomb Repressive Complex 2 (PRC2). In *Drosophila melanogaster*, these complexes are targeted to specific genomic loci primarily through cis-regulatory DNA sequences termed Polycomb response elements (PREs) [16]. In the classical hierarchical model of Polycomb repression, PRC2 and PRC1 act cooperatively to establish and maintain transcriptional silencing. PRC2 deposits the repressive histone mark H3K27me3, which facilitates the recruitment and stabilization of PRC1 at target loci [14]. PRC1 subsequently consolidates the repressed chromatin state through chromatin compaction and additional repressive chromatin modifications [16], ultimately restricting transcriptional initiation and enabling the stable inheritance of gene silencing across cell divisions. However, accumulating evidence indicates that PcG regulation involves reciprocal interactions between PRC1 and PRC2, diverse accessory factors, and context-dependent recruitment mechanisms [17,18,19,20]. In addition to canonical PRE-mediated targeting, PcG complexes can be recruited through alternative pathways involving DNA-binding factors, chromatin features, non-coding RNAs and developmental cues, highlighting the mechanistic plasticity of Polycomb regulation [21,22,23]. Their activities are indispensable for maintaining cell identity [24], sustaining stem cell self-renewal [25], coordinating body axis patterning [26], and directing organogenesis [27], thereby ensuring the faithful execution of developmental programs [6].

In *Drosophila melanogaster*, PcG proteins were initially identified through classical genetic screens based on homeotic transformations, including the characteristic ectopic formation of sex combs that gave the family its name [28,29]. Subsequent studies of the Bithorax complex (BX-C) demonstrated that PcG genes function as global epigenetic regulators that maintain Hox gene repression and preserve segmental identity during development. These discoveries established *Drosophila* as the foundational model for understanding Polycomb-mediated epigenetic memory [30]. Since then, the continued importance of *Drosophila* in PcG research is supported by its unique combination of experimental advantages. Advanced genetic tools, including GAL4/UAS, FLP–FRT and CRISPR/Cas9 systems, enable precise manipulation of PcG genes and regulatory elements with high spatial and temporal resolution [31,32,33]. Furthermore, the compact organization of *Drosophila* Hox clusters, together with the early discovery and extensive characterization of Polycomb response elements (PREs), has provided unparalleled opportunities to dissect the principles of Polycomb recruitment, chromatin domain formation and epigenetic inheritance [16,34]. PRE-associated DNA-binding factors, including Pho, and their interactions with PRC1 and PRC2 have established a mechanistic framework that continues to guide studies of Polycomb regulation across metazoans [35].

In this review, we summarize recent advances in the molecular mechanisms of PcG proteins and discuss their multifaceted roles throughout *Drosophila* development, with particular emphasis on the composition and regulation of Polycomb complexes and their functions in developmental gene control. By integrating current findings from *Drosophila* studies, we aim to provide a comprehensive overview of understanding Polycomb mediated epigenetic regulation and to highlight future research directions.

## 2. Molecular Mechanisms of Polycomb Function

### 2.1. Composition of PcG Complexes

PcG proteins function through the assembly of multisubunit complexes mediated by extensive protein–protein interactions. The coordinated activities of individual subunits within these complexes enable precise spatiotemporal control of target gene silencing. Early studies of Polycomb-mediated regulation in *Drosophila* focused primarily on the two core Polycomb repressive complexes, PRC1 and PRC2. With continued advances in biochemical purification methods and proteomic technologies, the molecular composition and functional classification of Polycomb complexes have been progressively refined, revealing a considerably greater degree of structural and functional diversity than previously appreciated.

**Figure 1:**
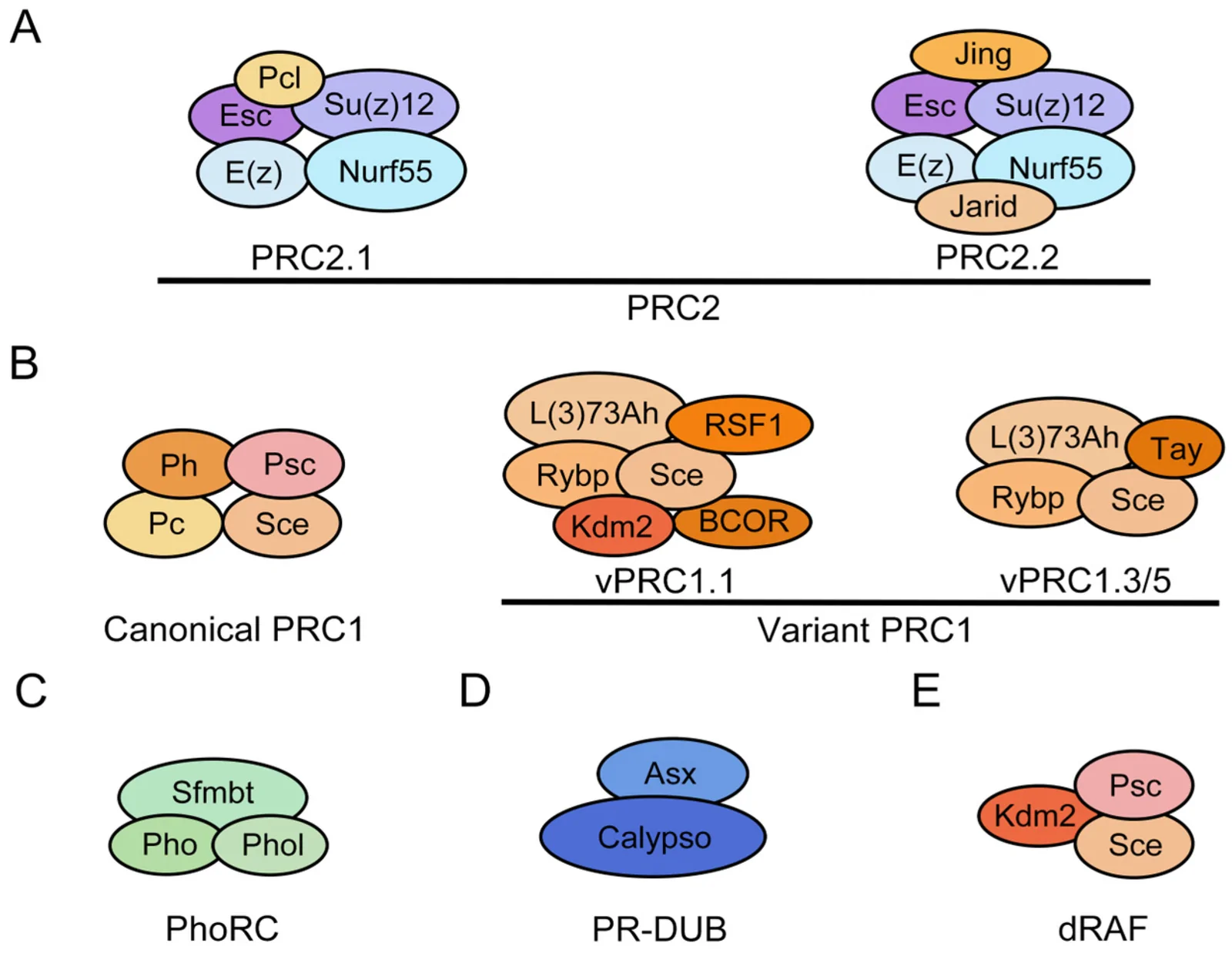
Diversity of Polycomb repressive complexes in *Drosophila melanogaster*. (**A**) PRC2 complexes. PRC2.1 (**left**) comprises the conserved catalytic core formed by enhancer of zeste (E(z)), suppressor of zeste 12 (Su(z)12), extra sex combs (Esc) and Nurf55, together with the Polycomb-like protein Polycomblike (Pcl). PRC2.2 (**right**) shares the same catalytic core but incorporates the accessory subunits Jarid2 and Jing. (**B**) PRC1 complexes. Canonical PRC1 (cPRC1) (**left**) is organized around the catalytic posterior sex combs (Psc)–sex combs extra (Sce) heterodimer and contains Polycomb (Pc) and Polyhomeotic (Ph) as structural subunits. Variant PRC1.1 (vPRC1.1) (**middle**) comprises a conserved L(3)73Ah–Rybp–Sce core together with the histone demethylase Kdm2. Variant PRC1.3/5 (vPRC1.3/5) (**right**) retains the L(3)73Ah–Rybp–Sce core and incorporates Tay. (**C**) PhoRC consists of Scm-related gene containing four MBT domains (Sfmbt) together with Pleiohomeotic (Pho) or Pleiohomeotic-like (Phol). (**D**) PR-DUB consists of Calypso and additional sex combs (Asx). (**E**) The dRAF complex comprises Psc, Sce and Kdm2.

PRC2 is built around a conserved catalytic core comprising enhancer of zeste (E(z)), suppressor of zeste 12 (Su(z)12), extra sex combs (Esc), and nucleosome-remodeling factor 55-kDa subunit (Nurf55) [36]. During specific developmental stages, Esc can be replaced by its paralog extra sex combs-like (Escl), giving rise to developmentally distinct PRC2 assemblies [37,38]. Based on the composition of their accessory subunits, *Drosophila* PRC2 can be further classified into two major subcomplexes (Figure 1A). PRC2.1 contains the accessory factor Polycomblike (Pcl), whereas PRC2.2 is characterized by the incorporation of Jumonji domain-containing protein 2 (Jarid2) and the zinc-finger protein Jing [12,39,40]. PRC2.1 is essential for the stable maintenance of high levels of H3K27me3 across Hox gene regions, whereas PRC2.2 plays a more heterogeneous or transitional rather than stable repressive role. The molecular diversity of PRC1 is similarly reflected in distinct canonical and variant assemblies (Figure 1B). For clarity, we distinguish canonical PRC1 (cPRC1) from variant PRC1 (vPRC1), which differ in composition, recruitment mechanisms and biological functions. cPRC1 comprises dRING/sex combs extra (dRING/Sce), Polycomb (Pc), Polyhomeotic (Ph), posterior sex combs (Psc), and suppressor of zeste 2 (Su(z)2) [30,41,42,43]. Psc and Su(z)2 are functionally redundant in *Drosophila* [44]. cPRC1 is primarily responsible for reinforcing transcriptional repression through chromatin compaction and the establishment of a repressive chromatin environment. By contrast, variant PRC1 (vPRC1) exhibits substantially greater compositional diversity [45]. Based on the current classification, *Drosophila* vPRC1 can be broadly divided into two major sub-complexes [40]. The vPRC1.1 complex consists of dRING/Sce, L(3)73Ah (the *Drosophila* orthologue of mammalian PCGF proteins), RYBP/YY1-binding protein (Rybp), lysine demethylase 2 (Kdm2), CG14073 (the BCOR orthologue), and CG8677 (the RSF1 orthologue). In contrast, the PRC1.3/5 complex is characterized by the incorporation of dRING/Sce, Rybp, L(3)73Ah, and Tay, the *Drosophila* orthologue of the mammalian UTS2, FBRS, and FBRSL1 proteins. In *Drosophila*, vPRC1 complexes can be recruited to chromatin through H3K27me3-independent mechanisms and exhibit diverse, context-dependent functions in gene regulation.

In addition to the core Polycomb complexes and their variants, several accessory PcG-associated complexes have been identified in *Drosophila*, further expanding the functional landscape of Polycomb-mediated gene regulation (Figure 1C–E). Among these, the Pho repressive complex (PhoRC) comprises Pleiohomeotic (Pho) or Pleiohomeotic-like (Phol) together with Scm-related gene containing four MBT domains (Sfmbt), and primarily facilitates the initial recruitment of Polycomb complexes to chromatin through Polycomb response elements (PREs) [35]. The Polycomb repressive deubiquitinase (PR-DUB) complex, consisting of additional sex combs (Asx) and Calypso, counteracts PRC1-mediated histone H2A ubiquitination by catalyzing H2A deubiquitination [36]. In addition, the *Drosophila* RING finger-associated factors (dRAF) complex, composed of dRING/Sce, Psc, and lysine demethylase 2 (Kdm2), possesses both H3K36 demethylaseassociated activity and H2A ubiquitin ligase activity, thereby providing an additional layer of epigenetic regulation [37]. The composition and major features of Drosophila Polycomb complexes are summarized in Table 1 [46].

An important refinement of the canonical Polycomb model has been the reassignment of sex comb on midleg (Scm). Scm should be distinguished from the core subunits of PRC1 and PRC2. Rather than forming an integral component of either complex, Scm acts as an independent bridging factor that promotes their spatial and functional coupling [47]. Its sterile alpha motif (SAM) domain mediates homo-oligomerization and interactions with PRC2, while Scm also directly associates with the PRC1 subunit Ph [48]. These multivalent interactions promote the spatial coupling of the two Polycomb complexes and contribute to the stabilizing Polycomb chromatin domains.

### 2.2. Polycomb Response Elements

Polycomb response elements (PREs) are specialized cis-regulatory DNA sequences that serve as nucleation sites for the recruitment of Polycomb complexes to target loci. PREs harbor multiple recognition motifs for sequence-specific DNA-binding proteins, including Pleiohomeotic/Pleiohomeotic-like (Pho/Phol) [49,50,51], GAGA factor (GAF) [52,53], Pipsqueak (Psq) [54,55], Sp1-like factor for pairing-sensitive silencing (Spps) [56], Combgap (Cg) [57], Zeste (Z) [58], Grainyhead (Grh) [59], dorsal switch protein 1 (Dsp1) [60], Adh transcription factor 1 (Adf1) [61], and crooked legs (Crol) [19]. Rather than acting independently, these factors cooperate through extensive functional interactions and partial redundancy to establish a robust recruitment platform that promotes the assembly of PRC1 and PRC2 at PREs [62,63]. Consequently, PREs function as specialized gene silencers that initiate and maintain Polycomb-mediated transcriptional repression.

PREs are highly dynamic regulatory elements that integrate developmental signaling with chromatin-based gene regulation. They respond to upstream developmental cues while functionally interacting with other cis-regulatory elements, particularly enhancers, to coordinate context-dependent gene activation or Polycomb-mediated silencing, ensuring the precise execution of developmental transcriptional programs [64]. Importantly, Polycomb-mediated repression is itself a highly dynamic process. PcG proteins can occupy PREs in both active and repressed chromatin states, and distinct PREs are deployed in a developmental stage- and tissue-specific manner throughout *Drosophila* development [65,66,67]. The regulatory activity of PREs is therefore not determined by any single DNA-binding factor but instead arises from the combinatorial and cooperative actions of multiple transcription factors. Consequently, the repertoire of factors required for PRE function can vary substantially among different genomic loci, reflecting the context-dependent nature of Polycomb recruitment and gene regulation [35,65,67,68]. This combinatorial regulatory strategy confers both flexibility and reversibility on Polycomb-mediated gene silencing, allowing transcriptional states to be dynamically modulated in response to developmental and environmental cues.

Beyond their linear sequence features, Polycomb response elements (PREs) contribute to the three-dimensional organization of the genome by acting as cis-regulatory anchors that promote chromatin looping and the formation of Polycomb domains [69,70]. Recent Hi-C, Micro-C and super-resolution imaging studies have revealed that these structures are dynamically established during development. In *Drosophila* embryos, Zelda-dependent active loops form before the mid-blastula transition (MBT), whereas repressive loops within Polycomb domains emerge after the MBT and involve GAGA factor and Polycomb proteins [23]. Disruption of PREs or PRE-mediated loop formation impairs Polycomb domain organization and reduces transcriptional repression, indicating that chromatin architecture actively contributes to Polycomb silencing rather than simply reflecting Polycomb occupancy. Super-resolution stimulated emission depletion (STED) microscopy of the *Drosophila* Hox clusters further showed that Polycomb foci are not homogeneous structures but contain dynamic PRC1 substructures termed PRC1 nanoglobules. PRC1 nanoglobules as dynamic Polycomb substructures that preferentially associate with PREs and contribute to Hox locus compaction [71]. PRE-mediated looping can also restrict enhancer–promoter communication, thereby insulating Polycomb-repressed genes from inappropriate activation [69]. Together with PRC2-mediated propagation of H3K27me3 and the retention of PcG proteins on mitotic chromosomes, these higher-order structures may facilitate the reestablishment of Polycomb domains after cell division [22,72]. Thus, PREs function not only as recruitment platforms but also as structural organizers that couple Polycomb occupancy, three-dimensional genome organization and epigenetic memory.

### 2.3. Canonical Polycomb Recruitment

**Figure 2:**
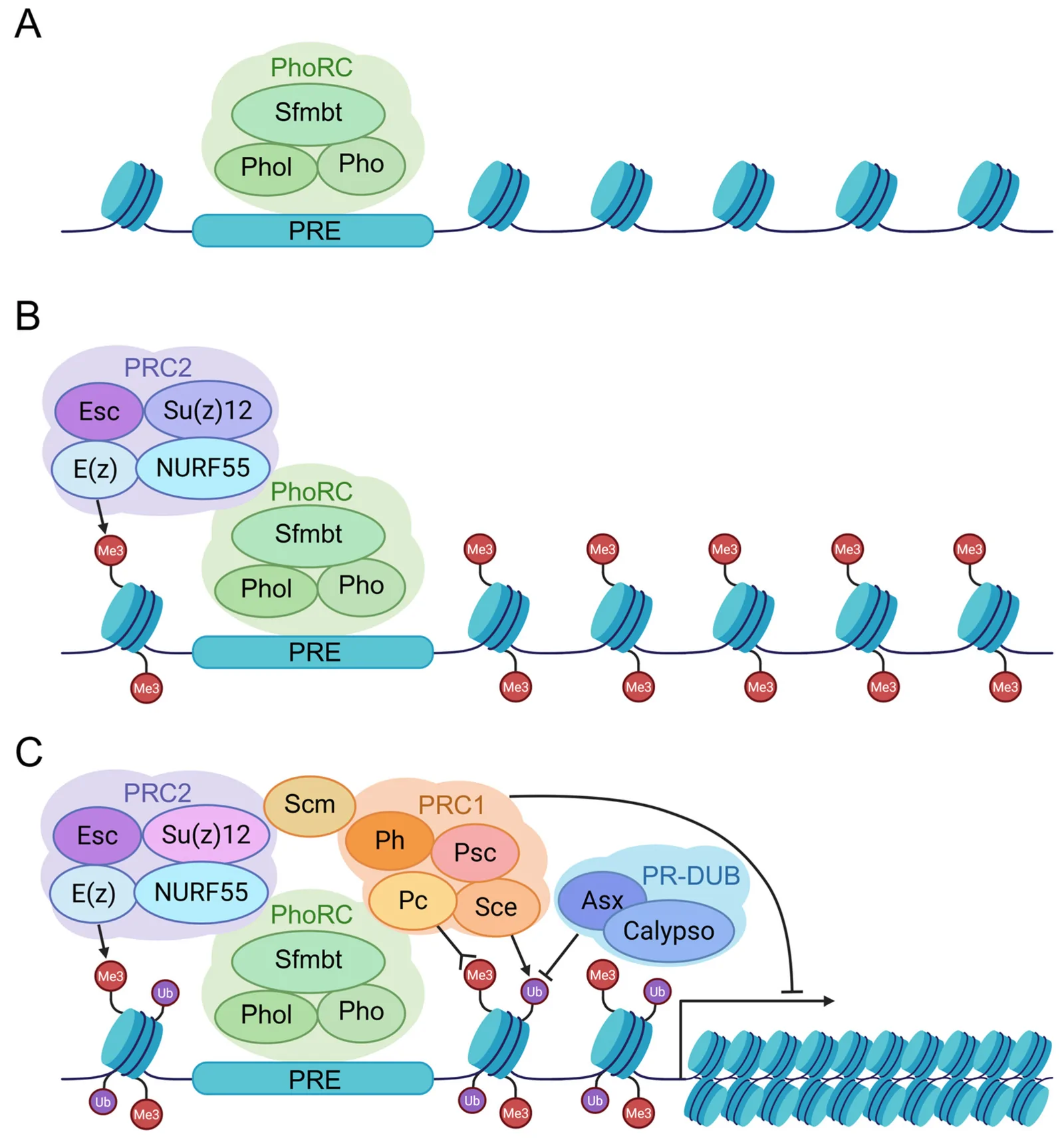
Stepwise assembly and spreading of Polycomb repression at Polycomb response elements (PREs). (**A**) PhoRC recognizes PRE DNA and recruits PRC2, resulting in local deposition of H3K27me3. (**B**) H3K27me3 is recognized by canonical PRC1 through Pc, promoting H2A monoubiquitination. Scm bridges H3K27me3-marked chromatin with PRE-bound Polycomb complexes to stabilize complex assembly. (**C**) Reciprocal positive-feedback between PRC2-mediated H3K27me3 and PRC1-mediated H2A monoubiquitination drives the spreading and maintenance of a compact Polycomb chromatin domain beyond the PRE. The PR-DUB complex counteracts PRC1-mediated H2AK118 ubiquitination via its deubiquitinase activity. Protein complexes, histone modifications and chromatin organization are shown schematically. The drawing was created using Biorender.com.

Studies of the *Ultrabithorax* (*Ubx*) *bxd* PRE and the *giant* (*gt*) PRE have established the canonical hierarchical model of Polycomb recruitment in *Drosophila* [65,73]. In this model, distinct Polycomb complexes are recruited in a coordinated and sequential manner to establish stable transcriptional repression, thereby forming an integrated epigenetic regulatory network (Figure 2). Polycomb recruitment is initiated by the PhoRC, which serves as the nucleation platform for Polycomb assembly [74]. Pho or its paralog Phol, the only PcG proteins possessing sequence-specific DNA-binding activity, recognize PREs in concert with additional DNA-binding factors. Subsequent recruitment of Sfmbt completes PhoRC assembly, which in turn recruits PRC2 through protein–protein interactions, thereby triggering the deposition of repressive histone modifications. Following its recruitment, PRC2 establishes the H3K27me3 landscape through the coordinated activities of its core subunits [36]. The catalytic SET domain of E(z) trimethylates histone H3 lysine 27 on nucleosomes surrounding PREs, generating the hallmark repressive chromatin modification that promotes subsequent PRC1 recruitment [75]. Esc specifically recognizes H3K27me3 through its WD40 repeat domain and allosterically enhances the catalytic activity of E(z), thereby establishing a reader–writer positive feedback loop that promotes the propagation and maintenance of H3K27me3 domains. Su(z)12 functions as a structural scaffold of PRC2, stabilizing complex integrity while facilitating nucleosome engagement [75,76]. Meanwhile, Nurf55 acts as a histone chaperone that supports efficient substrate recognition and histone modification by PRC2. Following PRC2-mediated deposition of H3K27me3, cPRC1 is recruited to these repressive chromatin domains through the coordinated actions of its constituent subunits. The chromodomain of Pc specifically recognizes H3K27me3, thereby directing the precise localization of cPRC1 to target loci [77,78]. The catalytic E3 ubiquitin ligase dRING/Sce subsequently monoubiquitinates histone H2A at lysine 118 (H2AK118ub) in *Drosophila* [79], reinforcing Polycomb-mediated transcriptional repression. Ph promotes higher-order chromatin organization through self-oligomerization, facilitating chromatin compaction and the formation of repressive Polycomb domains [80]. Meanwhile, Psc functions as a structural scaffold, maintaining the integrity and stability of the cPRC1 complex [16]. Collectively, the coordinated activities of Polycomb complexes establish a compact and transcriptionally repressive chromatin environment that restricts promoter accessibility and reinforces stable gene silencing. This repressive chromatin state further facilitates the recruitment and retention of additional PRC1 and PRC2 complexes, establishing a self-reinforcing regulatory circuit that propagates and stabilizes Polycomb-mediated repression [16,81].

Furthermore, although H2AK118ub facilitates the establishment of Polycomb-mediated repression, its abundance must be tightly controlled to preserve appropriate chromatin architecture. The PR-DUB complex counterbalances PRC1-mediated H2AK118 ubiquitination through its deubiquitinase activity, maintaining histone ubiquitination homeostasis and fine-tuning the degree of chromatin compaction. Importantly, the Polycomb-catalyzed histone modifications H3K27me3 and H2AK118ub constitute heritable epigenetic marks that are faithfully propagated during DNA replication and chromatin reassembly, enabling the maintenance of transcriptional states through successive cell divisions [82]. By preserving these chromatin states over developmental time, Polycomb complexes provide the molecular basis for epigenetic memory, thus ensuring the stable preservation of cell identity throughout development [83,84].

### 2.4. Non-Canonical Polycomb Regulation

Although the canonical model provides an important framework for understanding Polycomb repression, accumulating evidence indicates that Polycomb recruitment and activity are considerably more flexible than a strictly hierarchical PRC2-to-PRC1 pathway would suggest. Rather than conforming exclusively to a canonical hierarchical pathway, the recruitment, assembly, and regulatory functions of PcG complexes are highly context dependent and vary among genomic loci.

One prominent feature of non-canonical Polycomb regulation is that individual complexes can function independently of the classical PRC2-to-PRC1 recruitment cascade. This flexibility is particularly evident at PREs, which are often nucleosome-depleted and therefore do not require pre-existing H3K27me3 for initial Polycomb recruitment. Consistent with this view, PRC1 or dRAF can promote subsequent PRC2 recruitment at some PREs, whereas other PREs can recruit PRC1 and PRC2 through partially independent mechanisms [85]. Functional studies further support this model. During *Drosophila* development, repression of the *Ubx* locus can persist after sustained PRC1 occupancy is lost, provided that the H3K27me3 domain remains sufficiently extensive. Gene reactivation occurs only when H3K27me3 levels decline below a critical threshold, indicating that PRC2-mediated H3K27me3 deposition can, in certain developmental contexts, maintain transcriptional repression independently of continuous PRC1 occupancy [82,86]. This principle is not restricted to *Drosophila*. In mammals, RYBP/YAF2-containing vPRC1 complexes can be recruited independently of H3K27me3 and establish repression through H2AK118ub and alternative recruitment pathways [87]. Together, these findings support a flexible model in which PcG complexes engage chromatin through multiple recruitment cues and can operate through partially independent pathways rather than following a strictly hierarchical sequence.

Beyond their canonical repressive functions, Polycomb complexes can also contribute to transcriptional activation in a context-dependent manner. This functional plasticity is determined by complex composition, histone modification crosstalk, developmental signalling and interactions with Trithorax group (TrxG) factors. Changes in PRC1 composition or associated cofactors can redirect its chromatin activity towards gene activation. For example, the Pc subunit can associate with distinct cofactors to redirect Polycomb activity from repression towards transcriptional activation, indicating that the functional output of a Polycomb complex is not determined solely by its core composition. Chromatin state provides an additional layer of regulation [88]. The coexistence of H3K27me3 with active marks such as H3K4me3 can establish bivalent or poised states at developmental genes, allowing premature transcription to remain suppressed while preserving the capacity for rapid activation [89]. Trithorax group (TrxG) factors can further antagonize Polycomb repression by promoting active histone modifications and opposing PRC2-mediated H3K27me3 [16]. Conversely, PRC1 can cooperate with TET proteins or TrxG-associated factors such as Fs(1)h and Enok/Br140 to promote the expression of specific developmental genes [90,91]. Polycomb activity is also responsive to extracellular developmental signals. In *Drosophila*, 20-hydroxyecdysone (20E) signalling through the EcR/USP nuclear receptor complex can remodel Polycomb-associated chromatin during metamorphosis, thereby facilitating the stage-specific activation of genes required for developmental transitions [92,93,94]. At PRE/TRE elements, PcG and TrxG factors may coexist rather than act through simple mutually exclusive binding. Their relative activities, together with the direction of non-coding transcription, can shift the regulatory state towards repression or activation [65,95,96]. Moreover, Polycomb-dependent three-dimensional genome organization can contribute to either outcome: PRC1-mediated promoter–enhancer contacts at the *dachshund* (*dac*) locus can promote transcription under specific regulatory conditions [97]. Thus, Polycomb regulation is better understood as a context-dependent regulatory network rather than a unidirectional repression pathway, in which recruitment mode, complex composition, chromatin state and developmental signalling collectively determine whether a target locus is stably repressed, maintained in a poised state or activated.

### 2.5. Integrating Canonical and Non-Canonical Polycomb Recruitment

The canonical and non-canonical pathways should therefore be viewed as complementary rather than mutually exclusive modes of Polycomb regulation. The canonical pathway provides a sequential framework in which PRE-bound factors promote PRC2 recruitment, H3K27me3 deposition and subsequent cPRC1 engagement. Non-canonical pathways introduce flexibility by allowing PRC1-first recruitment, H3K27me3-independent targeting, complex-specific maintenance of repression and, in selected contexts, Polycomb-associated transcriptional activation. These pathways can operate independently or converge at the same locus, with their relative contribution determined by the underlying DNA elements, chromatin state, developmental stage and composition of PcG complexes. Thus, Polycomb recruitment is best understood as a context-dependent and interconnected regulatory network, rather than a fixed linear cascade. The major Polycomb recruitment pathways and their key features are summarized in Table 2 [65,73,82,85,86,87,88,89,97].

## 3. Polycomb Function in Embryonic Development

### 3.1. Maternal Polycomb Establishment Before ZGA

During early *Drosophila* embryogenesis, maternal Polycomb complexes establish a repressive chromatin state before widespread zygotic transcription. Maternal PRC2 deposits H3K27me3 at developmental loci, preventing premature activation of Hox and other developmental genes, whereas maternal cPRC1 promotes chromatin compaction [73,98,99,100]. Disruption of maternal Polycomb inheritance causes ectopic Hox expression and embryonic lethality [84,98].

This maternal state is actively remodeled during the maternal-to-zygotic transition (MZT). PR-DUB removes maternally deposited H2AK118ub, with Calypso counteracting maternal PRC1 activity and facilitating zygotic transcription [101,102,103]. Loss of Calypso causes persistent H2AK118ub, impaired zygotic transcription, and blastoderm arrest [101,102,103,104,105]. Thus, maternal Polycomb repression is reset before zygotic genome activation (ZGA).

### 3.2. Zygotic Polycomb Reprogramming During and After ZGA

Following the MZT, Polycomb regulation shifts from maternal inheritance to zygotically controlled repression. At nuclear cycle 14 (NC14), the pioneer factor Zelda renders a subset of PREs competent for Polycomb recruitment, promoting PRC2 nucleation and H3K27me3 deposition [106]. This transition coincides with degradation of maternally deposited *E(z)* mRNA [107]. During the syncytial blastoderm stage, PRC2-dependent H3K27me3 maintains repression of the gap gene *gt* [73], whereas by embryonic stage 14, PRC1 can maintain *tailless* (*tll*) silencing independently of PRC2 [108]. Polycomb also maintains Hox expression boundaries along the anterior–posterior axis; its loss causes ectopic Hox expression, homeotic transformations, and embryonic lethality [65,109,110,111]. Early H3K27me3 domains are progressively resolved along the axis in parallel with Hox activation [112,113]. In addition, H3K27me3-enriched domains associate with HP1a-marked heterochromatin to form topological domains that limit inappropriate interactions between repressed and active loci [114].

Together, these findings show that embryonic Polycomb regulation is dynamically remodeled from maternal repression to zygotically established and spatially resolved chromatin states, integrating histone modification, complex-specific recruitment, and three-dimensional genome organization.

## 4. Polycomb Function in Post-Embryonic Development

### 4.1. Larval Growth and Cell Proliferation

During larval development, Polycomb complexes preserve transcriptional memory and maintain Hox repression as tissues proliferate and differentiate. *E(z)* and *Su(z)12* remain at low but persistent levels, whereas *Esc* declines after embryogenesis and is progressively replaced by *Escl*, reflecting developmental remodeling of PRC2 [37,38,82,107,115]. Polycomb activity is also tissue specific during larval development. E(z) is enriched in the nervous system, intestinal epithelium, and gonads, where PRC2 supports the balance between stem-cell self-renewal and differentiation [25,116,117]. In the central nervous system (CNS), PRC2 prevents ectopic Hox activation and premature neuroblast differentiation [118]. Thus, PcG complexes coordinate developmental identity with tissue growth and homeostasis.

Polycomb complexes directly regulate larval growth and cell-cycle progression. E(z)-dependent repression of 4E-BP promotes protein synthesis and growth in imaginal discs [119]. PRC1 subunits Pc and Ph repress *Cyclin A* (*CycA*), while Polycomb-dependent control of *Cyclin D* (*CycD*) helps coordinate G1/S progression and tissue growth [6,120,121].

Notably, transient depletion of *Ph* during the first larval instar causes persistent hyperproliferation, loss of epithelial polarity, and impaired differentiation in the eye imaginal disc, and these defects are not rescued by restoring *Ph* expression [122]. This finding highlights the importance of Polycomb-mediated epigenetic memory in maintaining long-term proliferative homeostasis.

### 4.2. Cell Fate Determination

During larval development, Polycomb complexes preserve lineage identity by restricting inappropriate cell-fate programs. Loss of core PcG components disrupts these constraints and can cause cell-fate reprogramming and homeotic transformations in imaginal tissues [123,124,125].

The plasticity of imaginal discs illustrates this function. JNK-mediated repression of *Pc* and *Ph* in the leg disc relieves repression of *vestigial* (*vg*) and *wingless* (*wg*), enabling leg-to-wing conversion [27]. Similarly, Pc depletion in the eye disc activates *Antennapedia* (*Antp*) and promotes eye-to-wing transformation, while Polycomb disruption in the antennal disc can induce alternative appendage identities [126,127,128,129,130]. Polycomb components also maintain spatial restriction of *Ubx* and other Hox genes in imaginal discs [44,98,107].

Polycomb complexes also regulate terminal cell-fate decisions. Loss of Sce, Scm, Pc, or related PcG components disrupts photoreceptor fate specification, including the R7-to-R8 fate switch in the developing eye [24].

### 4.3. Metamorphosis and Tissue Remodeling

Larva-to-pupa transition requires extensive transcriptional reprogramming driven by 20-hydroxyecdysone (20E) and juvenile hormone (JH) [131,132,133]. Polycomb complexes contribute by regulating ecdysone biosynthesis and hormone-responsive transcription. In the prothoracic gland, PRC2 represses *Hairy* to facilitate ecdysone production, while Schlank cooperates with Polycomb to promote the H3K27ac-to-H3K27me3 transition during pupal development [134,135,136]. Polycomb-mediated chromatin organization also restricts premature activation of metamorphosis-associated genes [137,138,139].

During metamorphosis, the 20E–Sox14 pathway induces *Mical* for axon pruning, while Polycomb maintains appropriate Hox repression. Loss of Ph causes *Abd-B* derepression and defective pruning; PRC1 preferentially represses *Abd-B* and *Scr*, whereas PRC2 preferentially targets *Ubx* and *abd-A* [138,140,141].

Polycomb regulation also contributes to epithelial morphogenesis. The PRC1 catalytic subunit dRING/Sce represses *Abd-B* to promote wing eversion and partial epithelial-to-mesenchymal transition of the peripodial epithelium; *Sce* depletion causes defective wing eversion and thoracic clefts [142].

## 5. Polycomb Function in Adult Physiology

### 5.1. Circadian Rhythms and Intestinal Homeostasis

Beyond their developmental functions, Polycomb complexes continue to regulate physiological homeostasis throughout adulthood. In *Drosophila*, they contribute to the maintenance of circadian rhythmicity, sleep homeostasis, and intestinal tissue integrity, demonstrating that Polycomb-mediated epigenetic regulation remains essential beyond development.

In clock neurons, Pc associates with regulatory regions of *period* (*per*), *timeless* (*tim*), and neuropeptide genes, and *Pc* depletion disrupts circadian rhythmicity and light entrainment [143,144]. Pc also promotes normal sleep duration and homeostatic rebound through neuronal regulation [145,146]. In the intestine, PRC2 maintains intestinal stem-cell identity by repressing differentiation-associated genes; its disruption causes abnormal proliferation and differentiation and compromises epithelial homeostasis [25,147].

### 5.2. Environmental Adaptation and Ageing

Polycomb-mediated chromatin remodeling also contributes to environmental adaptation and ageing. Changes in PcG activity alter stress responses, metabolic adaptation, and age-associated transcriptional programs.

Partial loss of E(z) derepresses genes including *Abd-B* and *Odc1*, increasing resistance to oxidative stress and starvation and extending lifespan [148]. Loss of Pc or Su(z)2 in the adult fat body likewise extends lifespan through partially distinct mechanisms [149]. Under low temperature, reduced repression of Polycomb targets is accompanied by a shift from H3K27me3 toward H3K4me3, supporting transcriptional acclimation [150].

In the ageing brain, miR-34 represses the PRC2.1-associated factors Pcl and Su(z)12, reducing global H3K27me3 levels. This remodeling is associated with a more youthful transcriptional state and reduced polyglutamine aggregation and neurotoxicity [151].

### 5.3. Male Germline Homeostasis and Spermatogenesis

In the adult *Drosophila* testis, Polycomb complexes regulate germline stem-cell maintenance, lineage progression, and spermatogenesis within the hub-associated niche [152,153].

PRC2 contributes to germline homeostasis through E(z)-dependent promotion of spermatogonial dedifferentiation and replenishment of the GSC pool [116]. In CySCs, coordinated PRC1–PRC2 activity maintains H3K27me3-dependent repression of *Abd-B*, supporting CySC and GSC identity and testicular homeostasis [154].

The Polycomb-associated factor Enhancer of Polycomb (E(Pc)) also has specialized roles in spermatogenesis. In CySCs, E(Pc) promotes differentiation by restraining JAK–STAT and EGF signaling [155]. In the germline, it promotes the mitosis-to-meiosis transition by repressing *Cyclin B* (*CycB*) and promoting *Bag-of-marbles* (*Bam*), and it cooperates with dTip60 to support DNA double-strand-break repair and germ-cell survival [156].

Together, these findings indicate that Polycomb-associated regulation supports male fertility by integrating stem-cell maintenance, lineage progression, cell-cycle control, and genome integrity.

### 5.4. Female Germline Development and Oogenesis

Polycomb-mediated regulation also plays indispensable roles during female germline development, although its molecular mechanisms differ substantially from those operating during spermatogenesis. In the adult ovary, germline stem cells (GSCs) generate differentiating cystoblasts that undergo four rounds of incomplete mitosis to produce a 16-cell cyst, from which a single cell adopts oocyte fate while the remaining cells differentiate into nurse cells.

PRC2 is the principal Polycomb complex implicated in oogenesis. Loss of E(z) dis-rupts H3K27me3 deposition at cell-cycle regulators such as *Cyclin E* (*cycE*) and *dacapo* (*dap*), impairing oocyte specification and causing female sterility [157]. PRC2 also regulates the balance between GSC self-renewal and differentiation: Pcl restrains differentiation programs in GSCs, whereas Scm promotes H3K27me3 establishment at differentiation-associated loci [158].

Notably, oogenesis exhibits a striking functional asymmetry between the two canonical Polycomb complexes. In contrast to the dominant role of PRC2, PRC1 is largely dispensable for oocyte specification, GSC maintenance, and differentiation. Consistent with this observation, H2AK118ub remains at very low levels during these developmental processes and contributes minimally to transcriptional repression [157,158].

In addition to the canonical Polycomb machinery, the Polycomb-associated factor Sfmbt performs a specialized function during meiosis. Sfmbt suppresses excessive expression of genes encoding components of the synaptonemal complex (SC), thereby preventing aberrant SC polymerization and ensuring proper SC disassembly and faithful meiotic progression [159].

## 6. Conclusions

**Figure 3:**
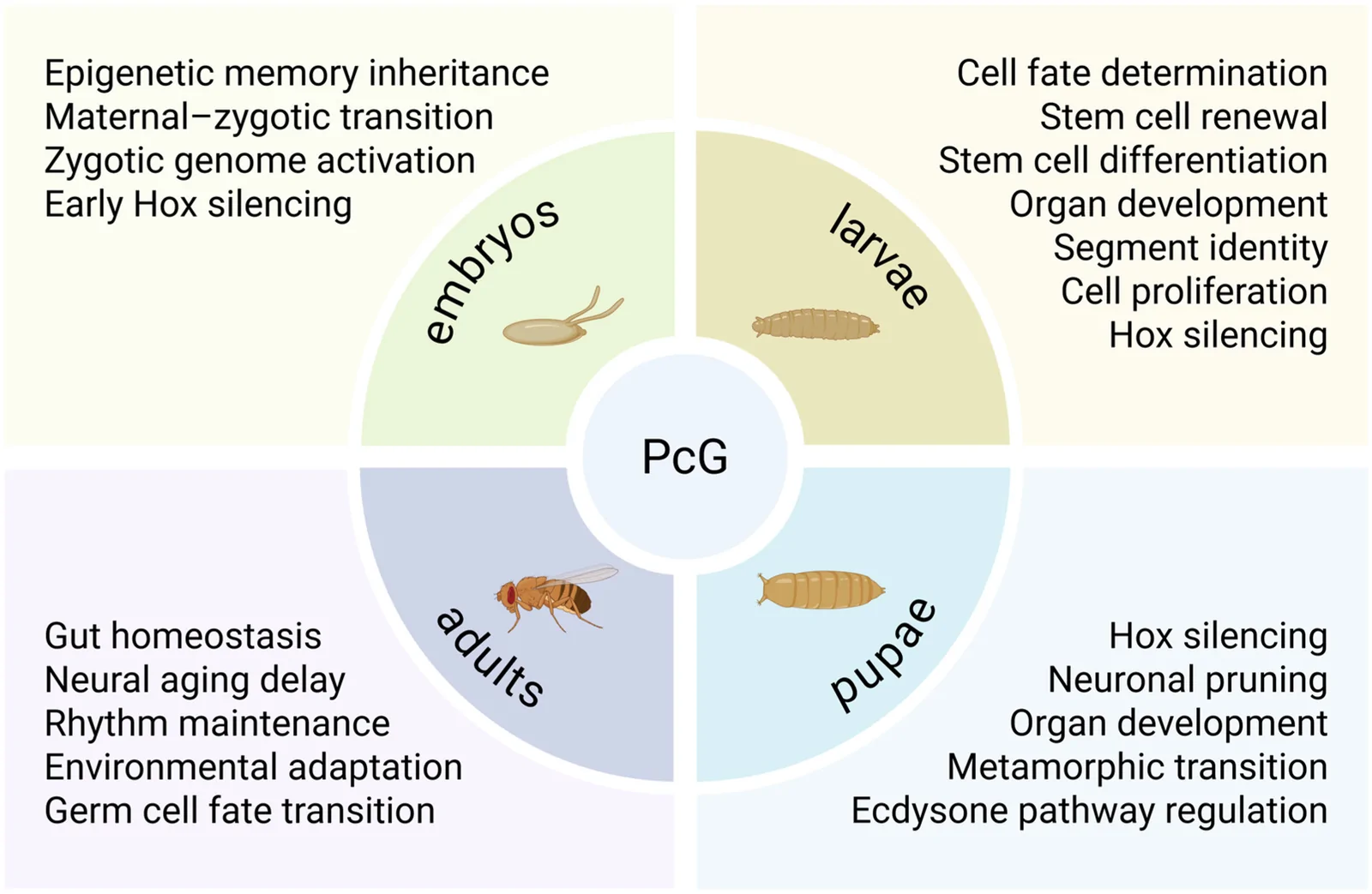
Stage-specific functions of Polycomb group proteins during *Drosophila* development. Polycomb group (PcG) proteins establish and maintain transcriptional repression through epigenetic mechanisms that operate throughout the *Drosophila* life cycle. During embryogenesis, PcG proteins contribute to epigenetic memory inheritance, maternal-to-zygotic transition, zygotic genome activation and early Hox silencing. In larvae, PcG activity regulates cell fate determination, stem cell maintenance, differentiation, organ development, segment identity and proliferation. During pupal metamorphosis, PcG proteins participate in Hox repression, neuronal remodelling, organ development and ecdysone pathway regulation. In adults, PcG-mediated regulation contributes to tissue homeostasis, neuronal ageing, circadian rhythm maintenance, environmental adaptation and germline fate transitions. The drawing was created using Biorender.com.

Polycomb group (PcG) proteins represent a highly conserved epigenetic regulatory system that occupies a central role in the developmental gene regulatory network of *Drosophila*. By assembling two core Polycomb repressive complexes (PRC1 and PRC2) together with multiple accessory modules, including PhoRC, PR-DUB, and dRAF, PcG proteins establish a multilayered regulatory architecture that integrates sequence-specific recruitment, histone modification, and higher-order chromatin organization. Through both canonical PRC2–PRC1 hierarchical recruitment mechanisms and non-canonical, complex-specific regulatory pathways, PcG systems ensure robust yet flexible control of transcriptional states. Importantly, PcG-mediated histone modifications can be faithfully propagated through cell division, thereby providing a molecular basis for stable epigenetic inheritance during development. Across the *Drosophila* life cycle, PcG complexes coordinate gene expression programs governing germ cell development and gametogenesis, embryonic segmentation, larval growth, larval-to-pupal metamorphosis, and adult physiological homeostasis (Figure 3). By modulating the spatiotemporal expression of key developmental regulators, particularly Hox genes, PcG proteins ensure accurate cell fate specification, organ identity maintenance, and developmental timing. This continuous regulatory input underscores the role of PcG systems as key determinants of developmental robustness and phenotypic stability in metazoans.

## 7. Perspectives

### 7.1. Fundamental Mechanisms of Polycomb Biology

Despite substantial advances in PcG research, important questions remain regarding how PcG complexes achieve functional diversity within species and how conserved regulatory principles are adapted across metazoans [160]. A major challenge is to determine how the composition and activity of distinct PcG assemblies are dynamically regulated across developmental stages, cell types and genomic contexts [161]. It is also unclear how the relative abundance and spatial distribution of H3K27me3, H2AK118ub and active chromatin marks shape transitions between stable repression, transcriptional poising and activation, and how PcG–TrxG interactions contribute to this plasticity [162,163,164]. Another important question is how PRE-associated chromatin contacts contribute to the establishment, maintenance and inheritance of Polycomb domains, particularly during cell division and developmental transitions [69,165,166]. Addressing these questions will require the integration of single-cell multi-omics, locus-specific epigenome editing and live-cell imaging with quantitative perturbation approaches. Such strategies should enable the dynamics of PcG complex assembly, chromatin modification and three-dimensional genome organization to be resolved at single-cell and single-locus resolution. Ultimately, defining how conserved Polycomb mechanisms are integrated with species- and context-specific regulatory inputs will be essential for understanding epigenetic memory, developmental plasticity and the evolution of chromatin-based gene regulation.

### 7.2. Human Health and Translational Medicine

Beyond their fundamental roles in development, the biological and translational significance of PcG dysregulation has attracted considerable attention. Aberrant Polycomb activity has been implicated in a wide range of physiological and pathological processes, including cellular aging [167,168], neurodevelopmental disorders [169], and tumorigenesis. Moreover, accumulating evidence highlights the therapeutic potential of targeting PcG pathways in diverse clinical settings, including adolescent depression [170], prostate cancer [171], ovarian cancer [172], and cardiac regeneration [173]. Several PcG components, including EZH2, CBX proteins and RNF2, have therefore emerged as potential therapeutic targets, with pharmacological strategies targeting Polycomb pathways being explored in cancers such as prostate, ovarian and hematological malignancies. These findings underscore the broad biomedical relevance of Polycomb-mediated epigenetic regulation.

An important future direction is to translate mechanistic insights from *Drosophila* into mammalian systems. In particular, non-canonical PcG activities and context-dependent activating mechanisms identified in flies may reveal new strategies for tissue regeneration and neural repair. At the same time, single-cell epigenomic approaches could help define cell-type-specific PcG dysregulation in patient tissues and identify disease-associated chromatin states. Integrating comparative Polycomb biology with functional epigenome editing and patient-derived models may ultimately enable more precise, context-specific therapeutic strategies targeting PcG pathways.

### 7.3. Agricultural and Insect Pest Management Applications

Beyond their fundamental roles in development, PcG proteins may also represent promising targets for sustainable insect pest management because of their essential functions in metamorphosis, tissue patterning and reproduction, particularly in light of the growing potential of RNA biopesticides based on spray-induced gene silencing. In the red flour beetle (*Tribolium castaneum*), RNA interference-mediated knockdown of *Pc* or *E(z)* induces homeotic transformations of adult head appendages into leg-like structures, posteriorization of the legs and wings, and multiple defects in appendage patterning and eye morphogenesis [174]. In the cricket *Gryllus bimaculatus*, RNAi of *E(z)* or *Su(z)12* produces profound embryonic patterning defects and lethality, further demonstrating the developmental importance of PcG function beyond holometabolous insects [175]. However, the feasibility of PcG-targeted RNAi for pest control remains strongly dependent on developmental stage, target specificity and delivery efficiency.

PcG-targeted RNAi is generally expected to be most effective during early larval stages, when developmental programs are highly active, whereas pupae and adults may show reduced sensitivity. This limitation is particularly relevant in Diptera, in which inefficient systemic uptake and processing of exogenous double-stranded RNA can substantially restrict RNAi efficacy compared with beetles and some lepidopterans [176]. Moreover, the evolutionary conservation of core PcG proteins raises concerns about potential effects on beneficial insects, including pollinators [177]. Future strategies should therefore combine pest-specific sequence selection with optimized delivery systems, such as tissue-specific or plant-mediated RNAi and improved dsRNA formulations, to enhance target specificity and systemic efficacy [178,179,180]. Comparative analysis of insect-specific or rapidly diverging PcG-associated subunits may further provide more selective targets than highly conserved core components. Integrating these approaches with comparative genomics and functional screening across pest and non-target species could facilitate the development of PcG-based biocontrol strategies that maximize pest suppression while minimizing ecological risks.

## Figures and Tables

**Table 1:** Core Polycomb group (PcG) complexes in *Drosophila*, their subunit composition, molecular functions, and characteristic structural domains.

Complex	Gene	Chromosome	Function	Characteristic Domains	Developmental Specialization
PRC1	*Pc*	3L	Recognizes and binds H3K27me3 marks; recruits additional PRC1 components	Chromodomain	cPRC1-specific; broadly expressed; peaks in early embryogenesis and declines thereafter; enriched in imaginal discs and ovarian germ cells.
	*Ph*	X	Chromatin compaction and polymerization; interacts with Scm to stabilize PRC1	FCS zinc finger, SAM	Broadly expressed; peaks at 0–12 h embryogenesis and declines sharply in adults; enriched in neuroectoderm, imaginal discs and ovarian germ cells.
	*Psc/Su(z)2*	2R	Stabilizes the PRC1 complex; facilitates H2AK118ub;	RING finger, RAWUL	Mutually exclusive cPRC1 subunits; Psc predominates maternally and early embryogenesis, peaks at 0–6 h and declines thereafter; Su(z)2 is low in embryos but induced in adult fat body; enriched in primordial germ cells and larval CNS.
	*Sce*(*dRing*)	3R	Catalytic subunit of PRC1; catalyzes H2AK118ub	RING finger, RAWUL	Shared cPRC1/dRAF catalytic subunit; supports early embryonic mitosis; peaks during pupal wing morphogenesis and is enriched in adult spermatocytes.
PRC1/2 Bridge	*Scm*	3R	Bridges PRC1 and PRC2; stabilizes PRC1 assembly	FCS/MYM zinc finger, SAM, MBT repeat, LED	Peaks during MZT and remains low thereafter; enriched in gonads, embryonic neuroectoderm and imaginal discs.
PRC2	*E(z)*	3L	Catalytic subunit of PRC2; catalyzes H3K27me3	SET, CXC, SANT1, SANT2	Peaks at 0–6 h embryogenesis and declines thereafter; enriched in neural tissues, intestine and germline stem cells.
	*Esc*	2L	Recognizes H3K27me3; stimulates the methyltransferase activity of E(z)	WD40 repeat	Embryo-specific PRC2 core subunit; replaced by Escl after embryogenesis; restricted to 0–12 h embryos and absent thereafter.
	*Su(z)12*	3L	Structural scaffold of PRC2; maintains complex stability and E(z) catalytic activity; facilitates PRC2 binding to nucleosomes and DNA	VEFS-box, zinc finger	Moderate embryonic expression; reduced during pupal metamorphosis and low in adults; enriched in larval prothoracic glands and adult brain.
	*Nurf55/Caf1-55*	2L	Promotes PRC2 association with nucleosomes; participates in chromatin assembly	WD40 repeat, CAF1B/HIR1 β-propeller	Broadly and constitutively expressed; mildly reduced after embryogenesis; enriched in ovarian germ cells and spermatogonia.
PhoRC	*Sfmbt*	2L	Recognizes mono- and dimethylated methyl-lysine residues	MBT repeat, FCS zinc finger, SAM	Broadly expressed in embryos; mildly reduced during pupal and adult stages; enriched in imaginal discs and germline stem cells.
	*Pho/Phol*	-	Binds PREs; recruits PhoRC and subsequently PRC1/PRC2 complexes	C2H2 zinc finger	Functionally redundant; Pho targets embryonic PREs, whereas Phol compensates in larval epithelium; both peak at 0–6 h; Pho is repressive, whereas Phol has dual functions.
PR-DUB	*Asx*	2L	Regulatory subunit of PR-DUB; activates the deubiquitinase activity of Calypso;	DEUBAD, PHD finger	Peaks during MZT and remains low thereafter; enriched in embryonic neuroectoderm and nuclear-localized in imaginal discs.
	*Calypso*	2L	Catalytic subunit of PR-DUB; deubiquitinates H2AK118ub	C12-family cysteine peptidase, ULD	Maternal mRNA supports early embryogenesis; downregulated in late pupae and mildly enriched in adult spermatocytes.
dRAF	*Sce*(*dRing*)	Same as PRC1			
	*Psc/Su(z)2*	Same as PRC1			
	*Kdm2*	3R	Demethylates H3K36me and H3K4me; facilitates PcG-mediated transcriptional repression	JmjC, F-box, zinc finger	Peaks during early embryogenesis and early pupal development; enriched in germ cells, larval body wall muscle and adult circadian neurons.

Data were compiled from FlyBase (release FB2026_02) [46].

**Table 2:** Distinct Polycomb recruitment and regulatory pathways in *Drosophila*.

Recruitment Mode	Histone Modification Dependence	Key Regulators	Major Physiological Contexts
Canonical PRE-dependent recruitment [65,73]	H3K27me3 and H2AK118ub	Pho/Phol, PhoRC, PRC2, cPRC1, Scm	Hox gene silencing, embryonic patterning and maintenance of developmental identity
PRC1-first recruitment [85]	Largely independent of pre-existing H3K27me3	PRE-binding factors, PRC1, dRAF	PRE-associated repression and locus-specific Polycomb domain establishment
PRC2-dependent maintenance/PRC1-independent repression [82,86]	Strongly dependent on H3K27me3; persists independently of sustained PRC1 occupancy after domain establishment	PRC2	Maintenance of established Polycomb domains and stable repression of selected developmental loci, including *Ubx*
H3K27me3-independent PRC1 recruitment [87]	Independent of H3K27me3	vPRC1, RYBP/YAF2-containing complexes	Context-dependent repression;
3D chromatin-mediated regulation [88,89,97]	Depends on chromatin architecture rather than a single histone mark	PRC1, PREs, architectural factors	Enhancer–promoter regulation, Polycomb domain organization and context-dependent activation/repression

## Data Availability

No new data were created or analyzed in this study. Data sharing is not applicable to this article.
